# The Little Professor and the Virus: Scaffolding Children’s Meaning Making During the COVID-19 Emergency

**DOI:** 10.3389/fpsyt.2020.00817

**Published:** 2020-08-13

**Authors:** Livio Provenzi, Elisa Baroffio, Susanna Ligabue, Renato Borgatti

**Affiliations:** ^1^ Child Neurology and Psychiatry Unit, IRCCS Mondino Foundation, Pavia, Italy; ^2^ AGRES Onlus, Massina di Cislago, Italy; ^3^ Centro di Psicologia e Analisi Transazionale, Milano, Italy; ^4^ Department of Brain and Behavioral Sciences, Università di Pavia, Pavia, Italy

**Keywords:** COVID-19, child, parent, emotion regulation, meaning making, pandemic, emergency

From the very first years of life, children try to make sense and meaning out of the different stimuli they receive from their physical and social caregiving environment. Eric Berne used to refer to this precocious intuition of the surrounding world as a Martian thinking, “the naivest possible frame of mind for observing Earthly happenings” (1). This way of thinking is typical of the psychological Child Ego state called the “Little Professor”, which harbors strategies that the child possesses for solving problems: intuition and prelogical thinking (2). So, with the “little professor” we can refer to the intuitive and creative—rather than logical—thought process that builds on the explorative attitude of young children and on their sensitivity to the surrounding environment (3). Of course, this meaning-making process is far from being a conclusive viewpoint on reality and it is critically affected by direct and indirect messages received from the adult caregivers, especially the parents.

As the coronavirus disease of the 2019 (Covid-19) is rapidly spreading worldwide, it’s reasonable to assume that even the children’s “Little Professor” is trying to develop a naïve theory of what is happening in the external world by incorporating different information sources. These may include the verbal messages (e.g., information, explanations) and the emotional expressions of their parents as well as delivered by the media and other adults. Notably, even when language comprehension is not fully developed, children are highly sensitive to the prosodic elements of human communications, including adults’ gesture and voice tone (4). For this reason, the parental scaffolding of meaning-making processes is crucial to help children cope with such unexpected and frightening events, disentangling unclear messages and making order within the large amount of potentially confusing information they receive about the Covid-19 epidemic (5). Indeed, children are now surrounded by adults wearing masks, talking to each other about the infection and they can perceive alarm and distress by looking and listening to them. They are supposed to change their habits: to respect strict hygiene standards and to remain at home with a dramatic reduction of physical social exchanges with peers. Additionally, they may have faced for the first time the loss of a significant person in their family. At the present moment, it is not easy for most parents to find verified and reliable information on the nature of this coronavirus as well as on the healthcare risks for themselves and their children (6). Scientists themselves are trying to understand the nature of the virus and they do not have conclusive estimations on the health-related risk as well as on the time course of the emergency (7).

This uncertainty—together with the lack of a specific and effective treatment for the Covid-19—can further feed the fears and the sense of vulnerability of citizens—both adults and children. In this context, whereas the healthcare policies adopted by different countries could help to contain and mitigate the infection spread, for most families they also represent severe restrictions to social relationships and habits (8). Previous research on the well-being of parents and children during and after healthcare emergencies suggest that both can develop post-traumatic stress symptoms (9). Increased prevalence of post-traumatic stress symptoms was reported in survivals of the SARS epidemic (10) and preliminary evidence of similar psychological effects are also emerging for the Covid-19 emergency (11). Notably, the stress perceived by parents may widely affect parenting behaviors (12) and the quality of parent-child interaction (13, 14). Neuroscientific (15) and epigenetic (16) evidence suggests that these stress-related parenting effects may have profound intergenerational consequences for children’s emotional and cognitive development (17–19). Thus, it is not surprising that the psychological consequences of Covid-19 emergency have been identified as the “second tsunami” of this unprecedented pandemic (20).

In sum, scaffolding children’s meaning-making process during the present pandemic is crucial to help them cope with the emergency situation and to avoid the overwhelming and traumatic effects of misleading or partial cognitive appraisal and emotional over-reactions. It is possible to identify different ways in order to create a safe environment in which parents and other adult caregivers (e.g., teachers, educators) can help young children to deal with the COVID-19 emergency. In this article, we would like to highlight four ways through which adults can guide their children through the meaning-making process: self-regulation, careful listening, simple talking, and playing and practicing together (**Figure 1**).

**Figure 1:**
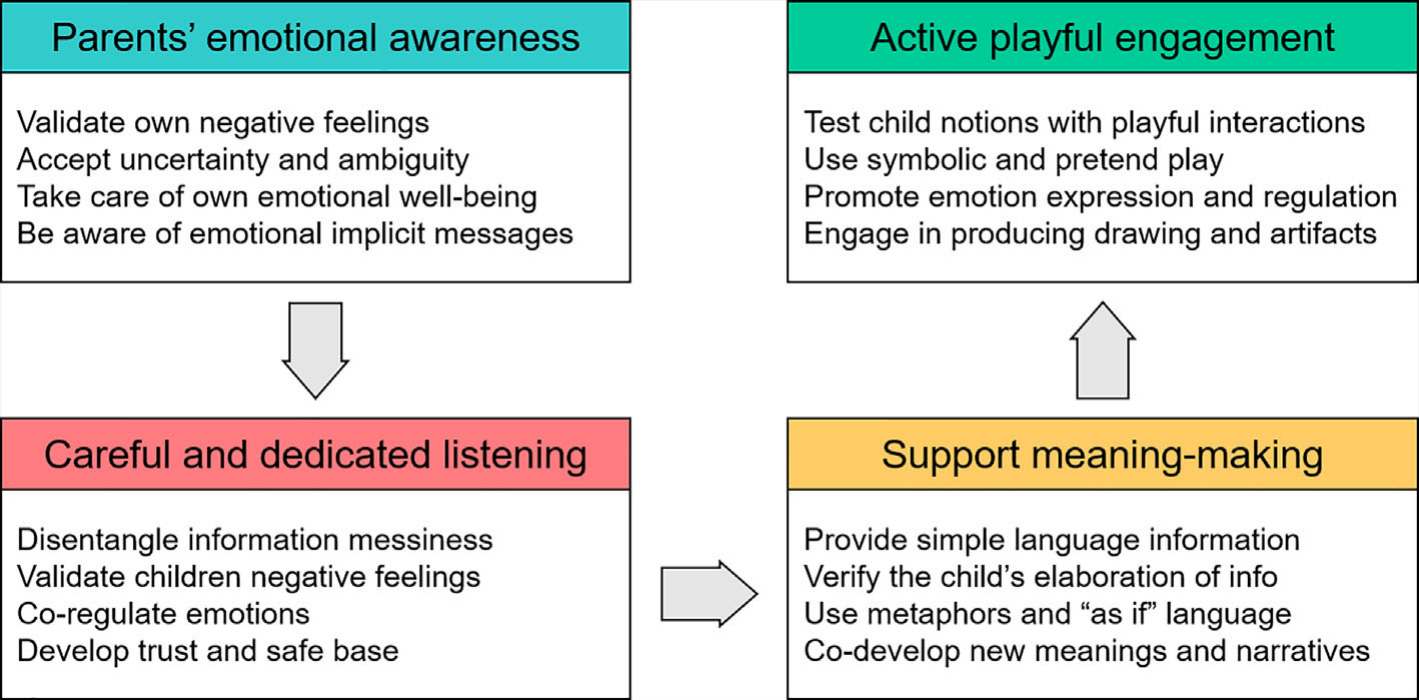
Schematic overview of parental actions aimed at supporting children’s meaning-making during the Covid-19 emergency.

First, parents should be in touch with their emotions and they are warranted to recognize, express, and regulate them in an adaptive way. Despite school-aged children may have a greater understanding of the verbal content of adults’ communications, infants are already sensitive to non-verbal cues such as looking, pointing, vocal tone, and other adults’ emotional and social expressions (21, 22). Even during preschool age, children could perceive the adults’ emotional state and they could respond consistently (23). Nonetheless, as especially young infants during the first two years of life may have only a partial access to the meaning of adults’ communications, their “Little professor”—who is constantly in search of coherent meanings—may be especially vulnerable to misinterpretations and pragmatic errors (24). In this context, infants may use the emotional expression of the caregivers to interpret the safety of ambiguous conditions (i.e., meaning-making) and to adopt consequent problem solving actions (e.g., coping strategies). The social referencing literature has largely provided examples of this by means of the so-called visual cliff experiment. In the visual cliff, infants move on a glass‐covered table divided into a shallow side under which a checkered pattern is placed right beneath the glass and a deep side under which a similar pattern is placed some distance below the glass, creating an apparent drop (25, 26). When mothers posed a happy expression, almost all infants crossed the cliff, whereas none of the infants who observed mothers’ fearful expression crossed, suggesting that at least from 12-month age infants resolve ambiguous conditions by integrating the parents’ emotional expressions in their implicit meaning-making (27). For this reason, caregivers should validate their own feelings of anxiety, fear, and worries and they should not neglect them dismissively. After all, it is in the reciprocal and mutual exchange of affective states that happen within the parent-child relationship, that children can develop appropriate and successful emotional regulation strategies and resilience to stress (28). Parents who are able to be in deep touch with their affective inner world, validating not only their positive emotional states but even depressive and anxious ones, can provide regulatory support and help their children deal with similar feelings, co-constructing with them instruments capable of adaptive emotional regulation (5). In other words, parents who let themselves express their real emotions will also grant the same permission to their young children. Reassuring children about the perceived alarm and risk for health can be successful only if it happens within a relationship characterized by genuine and open sharing of affective states.

Second, adults that do not neglect their own emotions can also promote a careful listening of children’s affective messages and communications. By supporting their child’s spontaneous emotional expression, adults can detect how the “Little Professor” in their child is trying to develop a coherent meaning of the situation. Indeed, previous research suggests that family-based narrative approaches provide a structured opportunity to elicit parents’ and children’s meaning-making, assemble divergent storylines into a shared family narrative, and thereby enhance members’ skills to cope with stressful and traumatic events developing hope and trust in family support (29). Careful and open listening by parents can allow children to freely express their feelings of fear and worries about the emergency within a relationship in which they may feel safe and protected (30). It should be highlighted that this personal creative and intuitive way of meaning making which is typical of the “little professor” is often limited in options (31). It provides emotional containment and protective survival strategies that require further scaffolding and permissions from parents to allow the emergence of more functional and adaptive coping strategies to face challenging life conditions. Additionally, far from hinder this intuitive thinking, adults can engage in a careful listening of children emotional world and they can understand which are the elements contributing to the emergent meaning-making process that they are developing (32). This is a crucial step for parents to provide further explanations to children and to promote a positive dialogue about the affective states and the cognitive representations arising from the lived experience of the Covid-19 emergency. Moreover, it should be important to note that this particular attitude to careful listening is warranted to continue across time as new information and knowledge can arrive to the children in different moments, thus requiring continuous interactive rearrangement and mutual refinement of the meaning-making process.

Third, when adults’ self-regulation is in place and careful listening is available for children, caregivers can now provide active contributions to the meaning-making process by using simple language. Avoiding complex concepts and explaining the emergency-related issues with age-appropriate words is crucial to clean up the messy ensemble of information to which the children are exposed (33). For example, receiving communications expressed in simple language can help the children to understand the Covid-19 symptoms, the risk factors and the appropriate behaviors needed to deal with the emergency. As the “Little Professor” use intuitive and analogic forms of representations, the use of metaphors, drawings and “as if” language can facilitate the integration of information by the child, stimulate curiosity and avoid the emergence of “monsters” or the persistence of scaring images in the meaning mindset of the child. Moreover, mother and fathers are encouraged to talk with their child together, as a way to communicate that the family as a system is coherent and to reinforce the strength of the messages. From this perspective, observing children’s spontaneous and subjective creations may allow the adults to monitor the meaning-making process that is unwinding within their inner world.

Fourth, the active engagement of parents during recreative activities can further scaffold children’s meaning-making during the Covid-19 emergency. Indeed, recreational activities represent the best secure setting in which parents and their children can share meanings about the actual emergency (34). During these moments, caregivers can enhance children’s intuitive and creative thinking, offering them coherent explanations about what is happening and directly co-constructing meanings and representations. As previously mentioned, the precocious experiences of parental holding and emotional regulation are key to make meanings about the physical, social, and psychological world the child is living in. For example, drawing and playing together allow parents and children to co-create a shared symbolic and analogic language through which a sensitive emotional education process is warranted to enhance children’s capacity to perceive, label, and differentiate among their own emotional feelings and affective states (35). By playing and practicing together, parents and children develop a shared grammar of meanings that will contribute to create a safe environment for psychological, emotional, and cognitive explorations later in life (36). In this crucial process, caregivers act like a mirror that may reflect and disentangle their child’s affective states. The current Italian context provides a clear example of this co-creation, which is the shared drawing of rainbows with the claim “Everything will be all right”. This symbolic creation highlights the importance to develop a common symbolism within the family that can also be shared on-line with peers, contributing to support hope and resilience for the future (37, 38).

In sum, in times of such an unprecedented global healthcare emergency, adults have the responsibility to take care and partner with children in producing integrated, coherent, and adequate meaning-making on the pandemic (39). In fact, young children create internal representations of their experiences of “being-with” the adult caregivers who support them to make sense about the surrounding environment (40). The cognitive and emotional appraisal of subjective experiences by the “Little Professor” allow the development of adaptive reactions to the situation and peculiar and subjective survival strategies. For this reason, by helping the present generation of children in dealing with the Covid-19 emergency, we hope adults can successfully contribute in nurturing a new generation of human beings that will share enhanced resiliency when faced with future unexpected and stressful events.

## Author Contributions

LP and EB conceived this work. EB, SL, and RB contributed to the drafting of the final version of the manuscript. All authors contributed to the article and approved the submitted version.

## Funding

This work was supported by funds from the Italian Ministry of Health (“Ricerca Corrente”, year 2020; “5 X 1000”, year 2017) to LP and RB.

## Conflict of Interest

The authors declare that the research was conducted in the absence of any commercial or financial relationships that could be construed as a potential conflict of interest.

## References

[1] Berne E What do you say after you say Hello! The psychology of human destiny. Grove Press: New York (1972).

[2] Cornell W De Graaf A Newton T Thunnissen M Into TA: A comprehensive textbook on transactional analysis. Karnac: London (2016).

[3] Alves TEC The Little Professor: Reflection on the Structure Development and Evolution of the Adult in the Child. Int J Trans Anal Res Pract (2019) 10:79–86. 10.29044/v10i2p79

[4] Bion RA Benavides-Varela S Nespor M Acoustic markers of prominence influence infants’ and adults’ segmentation of speech sequences. Lang Speech (2011) 54:123–40. 10.1177/0023830910388018 21524015

[5] Provenzi L Tronick E The power of disconnection during the COVID-19 emergency: From isolation to reparation. Psychol Trauma (2020) 12:S252–4. 10.1037/tra0000619 PMC8767960 32510232

[6] Chen Q Min C Zhang W Wang G Ma X Evans R Unpacking the black box: How to promote citizen engagement through government social media during the COVID-19 crisis. Comput Hum Behav (2020) 110:106380. 10.1016/j.chb.2020.106380 PMC7151317 32292239

[7] Remuzzi A Remuzzi G COVID-19 and Italy: what next? Lancet (2020) 395:1225–8. 10.1016/S0140-6736(20)30627-9 PMC7102589 32178769

[8] Graffigna G Barello S Savarese M Palamenghi L Castellini G Bonanomi A Measuring Italian Citizens′ Engagement in the First Wave of the COVID-19 Pandemic Containment Measures A Cross-sectional Study. MedRxiv (2020). 10.1101/2020.04.22.20075234 PMC7485890 32915822

[9] Cobham VE McDermott B Haslam D Sanders MR The role of parents parenting and the family environment in children’s post-disaster mental health. Curr Psychiatry Reps (2016) 18:53. 10.1007/s11920-016-0691-4 27086314

[10] Mak IWC Chu CM Pan PC You MGC Ho SC Chan VL Risk factors for chronic post-traumatic stress disorder (PTSD) in SARS survivors. Gen Hosp Psychiatry (2010) 32:590–8. 10.1016/j.genhosppsych.2010.07.007 PMC7132390 21112450

[11] Yuan R Xu QH Xia CC Lous CY Xie Z Ge QM Psychological status of parents of hospitalized children during the COVID-19 epidemic in China. Psychiatry Res (2020) 288:112953. 10.1016/j.psychres.2020.112953 32302814 PMC7153530

[12] Smith CL Stephens A Maternal stress and sensitivity: Moderating effect of positive affect. Parenting (2018) 18:1–8. 10.1080/15295192.2018.1405699

[13] Zietlow AL Nonnenmacher N Reck C Ditzen B Müller M Emotional stress during pregnancy–associations with maternal anxiety disorders infant cortisol reactivity and mother-child interaction at pre-school age. Front Psychol (2019) 10:2179:2179. 10.3389/fpsyg.2019.02179 31607996 PMC6773887

[14] Provenzi L Brambilla M Scotto di Minico G Montirosso R Borgatti R Maternal caregiving and DNA methylation in human infants and children: Systematic review. Genes Brain Behav (2020) 19:e12616. 10.1111/gbb.12616 31622002

[15] Enlow MB Egeland B Carlson E Blood E Wright RJ Mother–infant attachment and the intergenerational transmission of posttraumatic stress disorder. Dev Psychopathol (2014) 26:41–65. 10.1017/S0954579413000515 24059819 PMC4145695

[16] Yehuda R Lehrner A Intergenerational transmission of trauma effects: putative role of epigenetic mechanisms. World Psychiatry (2018) 17:243–57. 10.1002/wps.20568 PMC6127768 30192087

[17] Chan JC Nugent BM Bale TL Parental advisory: maternal and paternal stress can impact offspring neurodevelopment. Biol Psychiatry (2018) 83:886–94. 10.1016/j.biopsych.2017.10.005 PMC5899063 29198470

[18] Hartzell G Stenson AF van Rooij SJ Kim YJ Vance LA Hinrichs R Intergenerational effects of maternal PTSD: Roles of parenting stress and child sex. Psychol Trauma (2020). 10.1037/tra0000542 PMC7343607 31916804

[19] Oh DL Jerman P Marques SS Koita K Boparai SK Harris NB Systematic review of pediatric health outcomes associated with childhood adversity. BMC Pediatr (2018) 18:83. 10.1186/s12887-018-1037-7 29475430 PMC5824569

[20] Dutheil F Mondillon L Navel V PTSD as the second tsunami of the SARS-Cov-2 pandemic. Psychol Med (2020), 1–2. 10.1017/S0033291720001336 PMC7198460 32326997

[21] Repacholi BM Meltzoff AN Olsen B Infants’ understanding of the link between visual perception and emotion: “If she can’t see me doing it she won’t get angry”. Dev Psychol (2008) 44:561–74. 10.1037/0012-1649.44.2.561 PMC2586888 18331144

[22] Striano T Reid VM Social cognition in the first year. Trends Cognit Sci (2006) 10:471–6. 10.1016/j.tics.2006.08.006 16942896

[23] Bariola E Gullone E Hughes EK Child and adolescent emotion regulation: The role of parental emotion regulation and expression. Clin Child Fam Psychol Rev (2011) 14:198–212. 10.1007/s10567-011-0092-5 21424275

[24] Topál J Gergely G Miklósi Á Erdőhegyi Á Csibra G Infants’ perseverative search errors are induced by pragmatic misinterpretation. Science (2008) 321:1831–4. 10.1126/science.1161437 18818358

[25] Möller EL Majdandžić M Bögels SM Fathers’ versus mothers’ social referencing signals in relation to infant anxiety and avoidance: a visual cliff experiment. Dev Sci (2014) 17:1012–28. 10.1111/desc.12194 24909521

[26] Sorce JF Emde RN Campos J Klinnert MD Maternal emotional signaling: Its effects on the visual cliff behavior of 1-year-olds. Dev Psychol (1985) 21:195–200. 10.1037/0012-1649.21.1.195

[27] Vandivier LE Hertenstein MJ Social referencing in infancy: Important findings and future directions In: Mohiyeddini C Eysenck M Bauer S, editors, Psychology of emotions, motivations and actions. Handbook of psychology of emotions (Vol. 1): Recent theoretical perspectives and novel empirical findings. Nova Science Publishers (2013). pp. 81–5.

[28] Tronick E DiCorcia JA The everyday stress resilience hypothesis: A reparatory sensitivity and the development of coping and resilience. Children Aust (2015) 40:124–38. 10.1017/cha.2015.11

[29] Saltzman WR Pynoos RS Lester P Layne CM Beardlsee WR Enhancing family resilience through family narrative co-construction. Clin Chi Fam Psychol Rev (2013) 16(3):294–310. 10.1007/s10567-013-0142-2 23797387

[30] Williamson V Creswell C Butler I Christie H Halligan SL Parental experiences of supporting children with clinically significant post-traumatic distress: A qualitative study of families accessing psychological services. J Child Adolesc Trauma (2019) 12:61–72. 10.1007/s40653-017-0158-8 32318180 PMC7163877

[31] Ligabue S Emozioni e copione di vita. Quaderni Di Psicologia Analisi Transazionale E Sci Umane (2011), 55–6.

[32] Banella FE Tronick E Mutual Regulation and Unique Forms of Implicit Relational Knowing. In: Early Interaction and Developmental Psychopathology. New York: Springer (2019). p. 35–53.

[33] Struik A The trauma healing story. Healing chronically traumatised children through their families/whanau. Austr NZ J Fam Ther (2017) 38:613–6. 10.1002/anzf.1271

[34] Denham SA Renwick SM Holt RW Working and playing together: Prediction of preschool social-emotional competence from mother-child interaction. Child Dev (1991) 62:242–9. 10.1111/j.1467-8624.1991.tb01528.x

[35] Ring K What mothers do: Everyday routines and rituals and their impact upon young children’s use of drawing for meaning making. Int J Early Years Educ (2006) 14:63–84. 10.1080/09669760500446416

[36] Bruschweiler-Stern N Lyons-Ruth K Morgan AC Nahum JP The foundational level of psychodynamic meaning: Implicit process in relation to conflict defense and the dynamic unconscious. Int J Psychoanalysis (2007) 88:843–60. 10.1516/T2T4-0X02-6H21-5475 17681896

[37] Masten AS Resilience theory and research on children and families: Past present and promise. J Fam Theory Rev (2018) 10:12–31. 10.1111/jftr.12255

[38] Tronick E Beeghly M Infants’ meaning-making and the development of mental health problems. Am Psychol (2011) 66:107–19. 10.1037/a0021631 PMC3135310 21142336

[39] Dalton L Rapa E Stein A Protecting the psychological health of children through effective communication about COVID-19. Lancet Child Adolesc Health (2020) 4:346–7. 10.1016/S2352-4642(20)30097-3 PMC7270522 32243784

[40] Provenzi L Scotto di Minico G Giusti L Guida E Müller M Disentangling the dyadic dance: Theoretical Methdological and Outcomes Systematic Review of Mother-Infant Dyadic Processes. Front Psychol (2018) 9:348. 10.3389/fpsyg.2018.00348 29615947 PMC5868133

